# Urinary tract infections and antimicrobial sensitivity among diabetic patients at Khartoum, Sudan

**DOI:** 10.1186/s12941-015-0082-4

**Published:** 2015-04-21

**Authors:** Hamdan Z Hamdan, Eman Kubbara, Amar M Adam, Onab S Hassan, Sarah O Suliman, Ishag Adam

**Affiliations:** Department of Biochemistry and Molecular Biology, Faculty of Medicine, Al-Neelain University, P.O. Box 12702, Khartoum, Sudan; Faculty of Pharmacy, Al-Yarmouk University College, Khartoum, Sudan; Faculty of Medical Laboratory, Omdurman Al-Ahlia University, Omdurman, Sudan; Department of Obstetrics and Gynecology, Faculty of Medicine, Khartoum University, P.O. Box 102, Khartoum, Sudan

**Keywords:** Diabetes, Urinary tract infection, Bacteriuria, *E. coli*, *K. pneumoniae*, Sudan

## Abstract

**Background:**

Patients with diabetes mellitus (DM) are more susceptible to urinary tract infection (UTI) than non-diabetics. Due to the emergence of multidrug resistant (MDR) uropathogenic strains, the choice of antimicrobial agent is restricted. This study investigated the epidemiology of UTI, antimicrobial susceptibility, and resistance patterns of bacterial isolates from adult diabetic patients.

**Methods:**

A cross-sectional study was conducted at Khartoum Hospital, Sudan during the period of March − September 2013. Consecutive patients (men and women) were approached to participate in the study, irrespective of UTI symptoms. Socio-demographic and clinical data were obtained from each participant using pre-tested questionnaires. Clean-catch, midstream urine samples were collected and cultured for UTI diagnosis and antimicrobial susceptibility. Symptomatic bacteriuria was defined as a positive urine culture (≥10^5^ colony-forming units [CFU]/mL of a single bacterial species) from patients with symptoms associated with UTI; asymptomatic bacteriuria was defined as a positive urine culture from patients without symptoms associated with UTI.

**Results:**

A total of 200 diabetic patients were enrolled, 121 (60.5%) men and 79 (39.5%) women; 193 (96.5%) had type II DM. The overall prevalence of UTI was 39 (19.5%). Among the total population, 17.1% and 20.9% had symptomatic and asymptomatic bacteriuria, respectively. According to multivariate logistic regression, none of the investigated factors (age, sex, type of DM and duration) were associated with UTI. The predominant isolates were *Escherichia coli* (22, [56.4%]), and *Klebsiella pneumoniae*, [9, (23%)]. Eight of 22 *E. coli*, four of nine *K. pneumoniae* and one of five *Enterococcus faecalis* isolates originated from symptomatic patients. Six, four, three, and two of 22 *E. coli* isolates showed resistance to ampicillin, co-trimoxazole, nitrofurantoin, and amoxicillin-clavulanic acid, respectively. Two, two, one and one of nine *K. pneumoniae* isolates were resistant to ampicillin, co-trimoxazole, cephalexin, and amoxicillin-clavulanic acid. All 22 *E. coli* isolates were sensitive (100%) to gentamicin and cephalexin. All nine *K. pneumoniae* were sensitive to gentamicin (100%) and 88.8% were sensitive to cephalexin.

**Conclusion:**

In Sudan, about one-fifth of diabetic patients have UTI. *E. coli* is the most frequent isolate followed by *K. pneumoniae*.

## Introduction

Diabetes mellitus (DM) is a worldwide health problem, with an expected prevalence of 593 million by 2035 [1]. In Sudan, the prevalence of DM is 2.6%, including patients with poor glycemic control and about 67% with long-term complications of DM [2,3]. Urinary tract infection (UTI) is the most common infection among patients with DM and is responsible for considerable morbidity, particularly if it is unrecognized or untreated [4,5]. In Ethiopia, the rates of symptomatic and asymptomatic bacteriuria among diabetic patients are an estimated 13.6% and 10.4%, respectively [6]. Risk factors for UTI among patients with and without DM have been identified e.g. obesity, female sex, and prostate syndrome in men [7,8]. Furthermore, glycosuria, low immunity, and bladder dysfunction, which are associated with DM, are considered particular risk factors for UTI [9,10]. *Escherichia coli* is the most commonly isolated organism in both diabetic and non-diabetic patients [11,12].

The prevalence of DM is increasing worldwide and the emergence of multi-drug-resistant (MDR) strains is escalating; hence, determining the prevalence of UTI among diabetic patients and investigating the sensitivity of bacterial isolates to antimicrobial agents is important for the epidemiologist, scientist, health planner, and clinician. To the best of our knowledge, there are no published data regarding the epidemiology of UTI among diabetic patients in Sudan. Thus, this study was conducted at the Khartoum Hospital, Sudan, to provide epidemiological data about UTI among diabetic patients in Sudan.

## Methods

A cross-sectional study was conducted at Khartoum Hospital during the period March − September 2013. The hospital, with a capacity of 208 beds, is the largest governmental and referral hospital in the Sudanese capital of Khartoum, which receives referral cases from outside and within Khartoum state. Consecutive male and female patients with type I or type II DM who attended the referral clinic were approached to participate in the study, regardless of the presence or absence of UTI symptoms. Exclusion criteria included pregnancy, known underlying renal pathology or chronic renal disease, or use of antimicrobial therapy during the previous month. After providing written informed consent, relevant clinical and socio-demographic characteristics were collected using pre-tested questionnaires. Every patient was asked about symptoms suggestive of UTI (e.g., urgency, dysuria, urinary frequency, loin pain, and nausea) and history of other medical disorders, such as hypertension and, for males, prostate enlargement. Each patient’s weight, height, and body mass index (BMI) were calculated, and hemoglobin and blood glucose were measured.

### Specimen collection and processing

Participants were asked to provide a midstream urine sample according to the clean-catch procedure. Samples were collected using a sterile container that was refrigerated (4°C), transported in an ice-pack to the medical laboratory, and processed within 1 hour of collection. Using a standard quantitative loop, urine samples (1 μL and 10 μL) were used to inoculate Cysteine lactose electrolyte deficient (CLED) agar (Oxoid, Basingstoke, UK), MacConkey, 5% Sheep Blood agar, and chromogenic UTI (Oxoid) agar plates. Plates were incubated for 24 h at 37°C and the outcome was judged as significant/non-significant growth, or contaminated (discarded). Significant bacteriuria was defined as urine culture plates showing ≥10^5^ colony-forming units (CFU)/mL of single bacterial species. Symptomatic bacteriuria was defined as significant bacteriuria in addition to symptoms related to UTI, while asymptomatic bacteriuria was defined as significant bacteriuria in the absence of UTI symptoms. MDR bacteria were defined as isolates resistant to ≥2 antimicrobial agents.

### Identification of species and antimicrobial susceptibility testing

Chromogenic culture plates were used for growth morphology, then isolates were Gram-stained and species confirmed by in-house biochemical testing [13]. Gram-negative organisms, e.g. *E. coli*, *Klebsiella pneumonia*, and *Proteus mirabilis*, were distinguished by microscopy. *E. coli* was identified as medium, pink-to-red colonies and confirmed by positive indole test, whereas *K. pneumonia* were large, pink-to-mauve colonies, which were confirmed by negative oxidase and indole tests. *P. mirabilis* was assessed as small pale-to-colorless colonies testing positive to indole and urease but negative to oxidase. *Enterococcus faecalis* was the only Gram-positive microorganism that was isolated and was identified by the presence of small, turquoise colonies with coccoid morphology, which tested negative for catalase and positive for bile esculin.

The disc diffusion method was used to determine the antimicrobial susceptibility of isolates. Colonies were suspended in normal saline to 0.5 McFarland standard, and using disposable sterile swabs, the suspensions were inoculated on Muller-Hinton agar (Oxoid) and incubated for 18–24 h, according to Clinical and Laboratory Standards Institute (CLSI) guidelines [14]. *E. coli* ATCC® 25922 and *E. faecalis* ATCC® 29212 were used as control strains. Antimicrobial susceptibility and resistance was determined by isolate growth zone diameter according to CLSI guidelines as shown in Table 1. All antibiotic discs were from Oxoid. Amoxicillin-clavulanic acid was prescribed for symptomatic patients as empirical treatment before culture results were obtained. All patients were requested to return for urine culture results after 2 days and their treatment was evaluated.

**Table 1:** **Antimicrobial specific disc content and inhibitory zone diameter tested against** ***Enterobacteriaceae*** **and** ***Enterococcus faecalis*** **bacteria**

**Organism/antimicrobial**	**Disk content**	**Zone diameter**	
***Enterobacteriaceae***		**R**	**S**
Ampicillin	10 μg	≥17 mm	≤13 mm
Gentamicin	10 μg	≥15 mm	≤12 mm
amoxicillin-clavulanic acid	20 μg/10 μg	≥18 mm	≤13 mm
Cephalexin	30 μg	≥18 mm	≤14 mm
co-trimoxazole	1.25/23.75 μg	≥16 mm	≤10 mm
Nitrofurantoin	300 μg	≥17 mm	≤14 mm
***E. faecalis***			
Ampicillin	10 μg	≥17 mm	≤13 mm
amoxicillin-clavulanic acid	20 μg/10 μg	≥18 mm	≤13 mm
Nitrofurantoin	300 μg	≥17 mm	≤14 mm
Ciprofloxacin	5 μg	≥21 mm	≤15 mm

*Enterobacteriaceae include; E. coli, K. pneumoniae, P. mirabilis.*

### Statistical analysis

Data were entered into the computer using Statistical Package for the Social Sciences software for Windows version 16.0 (SPSS Inc., Chicago, IL, USA) and double-checked before analysis. Means and proportions of the socio-demographic and clinical characteristics were calculated and compared between the culture-positive and -negative groups using student *t* and ***X***^***2***^ tests, respectively. Univariate and multivariate analyses were used for the culture-positive group as dependent variables, and socio-demographic (age, sex and BMI) and clinical (duration of DM, type of DM, history of UTI, dysuria, urgency, hemoglobin and blood glucose levels) variables as independent parameters. Probability values of <0.05 were considered as statistically significant for all results.

## Results

Two hundred patients with DM were recruited; seven (3.5%) had type I and 193 (96.5%) had type II DM. There were more males than females, 121 (60.5%) vs. 79 (39.5%). Among the 200 diabetic patients, 76 (38%) had symptoms suggestive of UTI. The overall prevalence of UTI was 19.5%.

The prevalence of bacteriuria among symptomatic and asymptomatic diabetic patients was 17.1% and 20.9%, respectively, with no significant between-group difference. Compared with 22.3% of males, only 15.1% of females had bacteriuria (*P* = 0.043). Mean ages (SD) of the bacteriuric and non-bacteriuric patients were 58.6 (9.9) and 57.9 (11.6) years, respectively, (*P* = 0.981). There was no significant difference in the socio-demographic and clinical data between bacteriuric and non-bacteriuric diabetic patients (Table 2).

**Table 2:** **Clinical characteristics of patients and factors associated with UTI among diabetic patients using univariate and multivariate analyses**

**Variables**	**Patients with Bacteriuria**	**Patients without Bacteriuria**	**Univariate analysis**			**Multivariate analysis**		
	***n*** **= 39**	***n*** **= 161**	**OR**	**95% CI**	***P***	**OR**	**95% CI**	***P***
Age, years	58.6(9.9)*	57.9(11.6)*	1.0	0.9-1.0	0.641	1.0	0.9-1.0	0.548
Male sex	27(69.2)#	94(58.3) #	1.6	0.7-1.9	0.216	1.4	0.5-3.3	0.452
Body mass index, kg/m^2^	25.4(6.1)*	25.9(5.8)*	0.9	0.8-1.1	0.771	0.9	0.8-1.03	0.291
Duration of diabetes, years	16.7(11.7)*	14.4(8.5)*	1.0	0.9-1.1	0.412	1.0	0.9-1.0	0.422
Type II diabetes	35(89.7) #	158(98.1) #	6.0	1.2-28.1	0.022	4.3	0.6-27.5	0.121
History of UTI	5(12.8) #	18(11.1) #	1.1	0.4-3.3	0.773	1.2	0.3-3.8	0.721
Hemoglobin, g/dl	13.4(1.0)*	13.4(1.5)*	1.0	0.5-1.7	0.905	0.9	0.9-1.0	0.501
Blood glucose level, mg/dl	174.1(54.2)*	161 (42.1)*	1.0	0.9-1.0	0.433	0.9	0.9-1.0	0.248
Dysuria	13(33.3) #	63(39.1) #	0.7	0.3-1.6	0.504	0.7	0.3-1.6	0.448
Urgency	6(15.3) #	11(6.8) #	2.4	0.1-13.8	0.094	1.5	0.4-6.1	0.497

Data were shown as mean (SD) * or *n* (%) # as applicable. Abbreviations: OR, Odds Ratio; CI, confidence interval; *P*, probability value. All variables in the univariate analyses were added to multivariate analysis.

### Risk factors for urinary tract infections

Using logistic regression, although univariate analysis showed an association between type of DM and UTI, multivariate analysis showed no association between type of DM and other investigated factors (age, sex, type of DM, duration of DM, symptoms and BMI) and UTI (Table 2).

### Bacterial isolates and susceptibility to antimicrobials

Thirty-four (87.1%) and 5 (12.8%) of the 39 isolates were Gram-negative and -positive bacteria, respectively. The predominant organism isolated was *E. coli* (22 isolates, 56.4%). Other isolates were *K. pneumoniae* (9, 23.0%), *E. faecalis* (5, 12.8%), and *P. mirabilis* (3, 7.6%).

### Antimicrobial susceptibility pattern of bacterial isolates

#### Gram-negative bacteria

Gram-negative bacteria (*E. coli*, *K. pneumoniae* and *P. mirabilis*) were tested against six antimicrobial agents: ampicillin, gentamicin, amoxicillin-clavulanic acid, cephalexin, co-trimoxazole, and nitrofurantoin. All isolates (100%) were susceptible to gentamicin. *E. coli* (the predominant isolate) and *P. mirabilis* were 100% susceptible to cephalexin. Moreover, 8 out of the 9*K. pneumonia* isolates were susceptible to cephalexin (Table 3).

**Table 3:** **Antimicrobial susceptibility pattern of Gram-negative and Gram-positive bacteria isolated from diabetic patients**

**Isolates**	**RXN**	**Antimicrobial agents (%)**						
		**AP**	**CN**	**A-C**	**CEX**	**SXT**	**NF**	**CIP**
*Gram-negative*								
*E. coli* (*n* = 22)	S	16 (72.7)	22 (100)	20 (90.9)	22 (100)	18 (81.8)	19 (86.3)	Nt
	R	6 (27.2)	-	2 (9)	-	4 (18.1)	3 (13.6)	Nt
*K. pneumonia* (*n* = 9)	S	7 (77.7)	9 (100)	8 (88.8)	8 (88.8)	7 (77.7)	9 (100)	Nt
	R	2 (22.2)	-	1 (11.1)	1 (11.1)	2 (22.2)	-	Nt
*P. mirabilis* (*n* = 3)	S	2 (66.6)	3 (100)	3 (100)	3 (100)	2 (66.6)	2 (66.6)	Nt
	R	1 (33.3)	-	-	-	1 (33.3)	1 (33.3)	Nt
**Total (** ***n*** **= 34)**	**S**	**25 (73.5)**	**34 (100)**	**31 (91.1)**	**33 (97)**	**27 (79.4)**	**30 (88.2)**	Nt
	**R**	**9 (26.4)**	**-**	**3 (8.8)**	**1 (2.9)**	**7 (20.5)**	**4 (11.7)**	Nt
Gram*-positive*								
*E.faecalis* (*n* = 5)	S	4(80)	Nt	5(100)	Nt	Nt	5(100)	4(80)
	R	1(20)	Nt	-	Nt	Nt	-	1(20)

Key: S, Sensitive; R, Resistant; −, zero; Nt, Not tested; AP, ampicillin; CN, gentamicin; A-C, amoxicillin-clavulanic acid; CEX, cephalexin; SXT, co-trimoxazole; NF, nitrofurantoin; CIP, ciprofloxacin.

#### Gram-positive bacteria

*E. faecalis* (n = 5) were the only Gram-positive bacteria isolated in this study. As assessed against four antimicrobial agents, ampicillin, amoxicillin-clavulanic acid, nitrofurantoin, and ciprofloxacin, *E. faecalis* isolates were 100% susceptible to amoxicillin-clavulanic acid and nitrofurantoin. Only 20% of the *E. faecalis* isolates were resistant to ampicillin and ciprofloxacin (Table 3).

#### Multi-drug-resistance patterns of the isolates

Multi-drug-resistance was observed in 11 (28.2%) of the total 39 isolates. Twenty-three percent (5/22) of the *E. coli* isolates showed multi-drug-resistance against two to four antimicrobial agents (Table 3). Twenty percent (1/5) of the *E. faecalis* isolates (the only Gram-positive bacteria), showed multi-drug-resistance against two antimicrobial agents (Table 4).

**Table 4:** **Figure of bacteria resistance to two, three and four antimicrobial agents**

**Antibiogram**			
**No. (%) of resistance**			
**Organism**	**R2**	**R3**	**R4**
**Gram-negative**			
*E. coli* (*n* = 22)	3 (13.6)	1 (4.5)	1 (4.5)
*K. pneumonia* (*n* = 9)	2 (22.2)	-	1 (11.1)
*P. mirabilis* (*n* = 3)	-	1 (33.3)	-
**Total (** ***n*** **= 34)**	5 (14.7)	2 (5.8)	2 (5.8)
**Gram-positive**			
*E. faecalis(n = 5)*	1 (20)	-	-

**R2–R4 =** number of antimicrobial agents to which a given isolate was resistant.

## Discussion

The main findings of the present study were that the prevalence of symptomatic, asymptomatic, and overall bacteriuria among diabetic patients was 17.1%, 20.9%, and 19.5%, respectively. *E. coli* was the most common organism isolated. The reported prevalence of symptomatic and asymptomatic bacteriuria in this study was higher than the 13.6% and 10.4% reported in Ethiopia, a neighboring country [6]. Sudanese diabetic patients have poor glycemic control, which may explain the high prevalence of UTI in this setting [2]. Poor control of DM increases the risk of UTI by 24% [15]. Generally, compared with non-diabetic patients, diabetic patients have a higher incidence of UTI and asymptomatic bacteriuria [16,17]. However, we found that the prevalence of symptomatic and asymptomatic bacteriuria among pregnant women in our previous work was 12.1% and 14.7% respectively [12]. It is worth mentioning that in the current study, significant bacteriuria was defined as 10^5^ CFU/mL regardless of patients’ symptoms. If a lower bacterial count, such as 10^3^ CFU/mL associated with patients’ symptoms had been used, then a higher percentage of symptomatic and asymptomatic bacteriuria would have been obtained.

In the current study, none of the investigated factors (patient age, duration and type of DM) were associated with the prevalence of UTI. Similar findings were reported for diabetic patients in Saudi Arabia [7]. Previous studies have shown that older age, duration of DM, and level of DM control are risk factors for UTI among diabetic patients [4,18]. Likewise, BMI, history of UTI, and sexual intercourse have been reported as independent risk factors for UTI among diabetic patients [7,19,20]. In this study, in line with one previous report [7], no association between duration of DM and UTI was observed.

Diabetic patients are at increased risk of infection in general and, in particular, to UTI [21]. The susceptibility of diabetic patients to UTI could be explained by diminished neutrophil response, lower urinary cytokines, and leukocyte concentrations, which might facilitate the adhesion of microorganisms to uroepithelial cells [16,22,23]. Interestingly, sexual intercourse was reported as a risk factor for UTI in women regardless of their DM status [24,25]. It is difficult to investigate sexual practice in this setting (due to cultural and traditional regulations); if this had been investigated, perhaps different results might have been obtained.

In this study, 63 (39.1%) patients had dysuria without bacteriuria. Other diseases, such as tuberculosis and sexually transmitted diseases (STDs), which were not investigated in this study, could explain the dysuria without bacteriuria. The current study showed that *E. coli* was the most common organism isolated from both symptomatic and asymptomatic patients, and it was resistant mainly to ampicillin, co-trimoxazole, nitrofurantoin, and amoxicillin-clavulanic acid. This is in line with reports from Ethiopia, Libya, and Kenya [6,11,26]. Furthermore, this is in agreement with a recent report from Ethiopia, where over 60% of the isolated urinary *E. coli* was resistant to ampicillin [6]. Emergence of uropathogenic MDR *E. coli* was previously reported among pregnant women in the same region [12]. However, increasing evidence shows an increase in strains of MDR *E. coli* in diabetic and non-diabetic [27,28]. Niranjan and Malini claim that DM *per se* is a risk factor for infection by MDR *E. coli* [29]. This report is contradicted by other studies [30,31]. In our study, there was no association between DM and the development of UTI, and the number of MDR *E. coli* strains was small. Patients’ geographical region, lifestyle and health care factors may possibly be related to MDR *E. coli* [32]. *K. pneumoniae* was the second most commonly isolated organism, which is in agreement with a recent report from Nepal [33]. However, this order of isolated microorganisms does not differ from that reported in non-diabetic patients [34].

## Conclusion

About one-fifth of diabetic patients had UTI in our study. In both symptomatic and asymptomatic diabetic patients with UTI in Sudan, *E. coli* was the most frequent isolate followed by *K. pneumoniae*. Multi-drug resistance was observed in 28.2% of the total isolates. Ninety-seven percent of the Gram-negative bacteria were sensitive to cephalexin, while all Gram-negative organisms showed 100% sensitivity to gentamicin.

### Ethics

This study was approved by Al-Neelain Research Ethics Review Board, Sudan.
